# The emerging role of DNA methylation in the pathogenicity of bacterial pathogens

**DOI:** 10.1128/jb.00108-25

**Published:** 2025-07-17

**Authors:** Ya-xuan Ma, Xiu-dan Wang, Xin-min Li

**Affiliations:** 1 Institute of chronic diseases, The Affiliated Hospital of Qingdao University, School of Basic Medicine, Qingdao University 12593, Qingdao, China; 2 State Key Laboratory of Marine Food Processing & Safety Control, College of Food Science and Engineering, Ocean University of China 12591, Qingdao, China; University of Southern California Los Angeles, Los Angeles, California, USA

**Keywords:** DNA methylation, pathogenicity, opportunistic pathogens, DNA methylation sequencing, virulence factor expression, antimicrobial resistance

## Abstract

Uncovering the mechanisms regulating the pathogenicity of bacterial pathogens can help improve diagnostic capabilities and aid the development of new drugs, both of which are crucial for reducing the burden caused by bacterial infections. In recent years, with advancements in third-generation sequencing technologies, increasing evidence has shown that DNA methylation plays a pivotal role in the pathogenicity of bacterial pathogens. We believe that the key DNA methyltransferases involved in pathogenicity represent promising targets for antimicrobial therapies and that the DNA methylation sites involved in bacterial pathogenicity are important biomarkers for diagnosing bacterial infections. In this review, we summarize the following topics: (i) methods for DNA methylation sequencing; (ii) the involvement of DNA methylation in antibacterial drug resistance; (iii) the influence of DNA methylation on the expression of bacterial virulence genes; (iv) the impact of DNA methylation on bacterial biofilm formation, adhesion, and motility; and (v) the role of DNA methylation in bacterial adaptation. We hope to provide insights into bacterial pathogenicity from the perspective of bacterial epigenetics.

## INTRODUCTION

Bacterial infection is the second leading cause of mortality globally, accounting for approximately one in eight deaths worldwide (1) and posing a threat to global public health. The development of efficient antimicrobial agents and the identification of specific biomarkers associated with bacterial pathogenicity are crucial steps toward addressing this global public health problem (2). Increasing evidence indicates that DNA methylation plays a critical role in the pathogenicity of bacterial pathogens.

DNA methylation was first observed in the restriction modification (R-M) system, which mainly protects bacteria from foreign DNA invasion (3). Most methyltransferases (MTases) are associated with the R-M system, but some conserved MTases, including Dam, Dcm, and CcrM, are independent of the R-M system (4). DNA methylation is a well-known epigenetic modification in bacteria, with N6-methyladenine (6mA), C5-methylcytosine (5mC), and N4-methylcytosine (4mC) being the three major types of DNA modification (5). Methylation at specific DNA sites may modify the curvature of DNA (6), affect its thermal stability, and ultimately influence the interactions between DNA and DNA-binding proteins (7). A variety of studies have demonstrated that DNA methylation regulates various cellular processes, including DNA replication initiation, mismatch repair, gene expression, and cell cycle progression.

With the advancement of techniques for detecting DNA methylation, methods such as single-molecule real-time (SMRT) sequencing, enzymatic methylation sequencing (EM-seq), whole-genome bisulfite sequencing (WGBS), nanopore sequencing, and other emerging technologies are being gradually applied to the study of DNA methylation in bacteria. Growing evidence has demonstrated that DNA methylation contributes to bacterial pathogenicity by regulating the formation of biofilms, adhesins, flagella (8), fimbriae (9), spores, and other virulence factors. Furthermore, evidence has shown that DNA methyltransferases (DNMTs) can influence the pathogenicity of several pathogens (10), including *Escherichia coli*, *Salmonella* spp., *Yersinia pseudotuberculosis*, and *Vibrio cholerae*. These studies reveal that some DNMTs are linked to specific phenotypes of pathogens. However, elucidating how key methylation sites are epigenetically regulated and identifying the related mechanisms within a single study remain challenging.

In this review, we address the following questions: How does DNA methylation impact the phenotypes of pathogens? Which specific methylation sites serve as critical regulatory elements of pathogenicity? What are the underlying mechanisms regulating virulence programming in bacterial pathogens? We focus on summarizing recent progress related to the epigenetic regulation of pathogenicity in bacterial pathogens, especially the role of DNA methylation in controlling drug resistance, survival, virulence factor expression, biofilm formation, motility, and other related processes. We believe that a systematic examination of the mechanisms involved in regulating bacterial pathogenicity will provide crucial targets for the development of antibacterial agents. Identifying key DNA methylation sites involved in bacterial pathogenicity can provide important biomarkers for monitoring and preventing bacterial infectious diseases.

## METHODS FOR DNA METHYLATION SEQUENCING

DNA methylation sequencing plays a critical role in characterizing the methylomes of bacterial pathogens and revealing the mechanisms that regulate the pathogenicity of bacterial pathogens. While much research on DNA methylation in eukaryotes has flourished over the past few decades, research advances related to bacterial DNA methylation have been relatively limited over the same period, mainly due to the lack of effective methods for prokaryotic DNA methylation sequencing.

**Fig 1:**
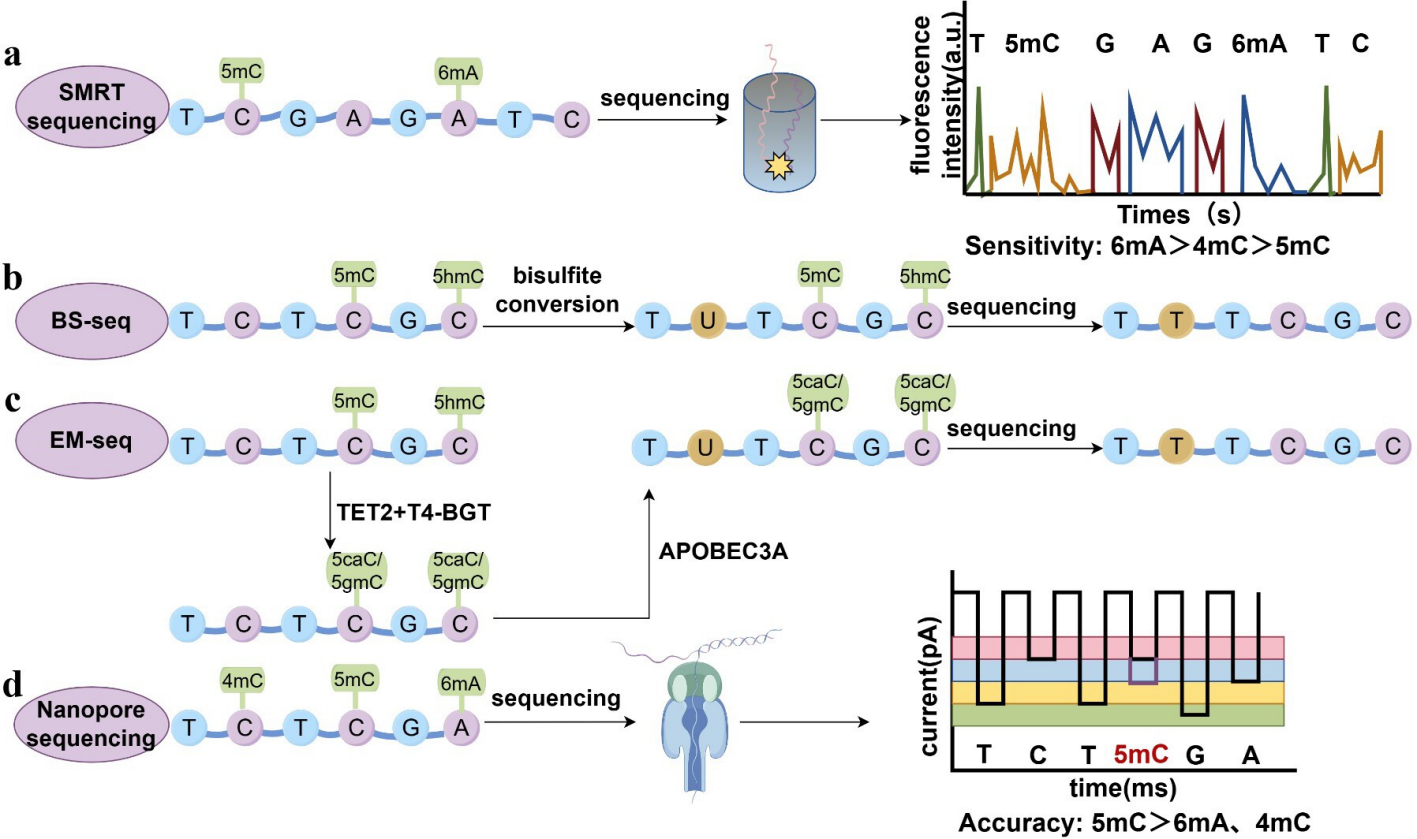
Principles of representative methods for DNA methylation sequencing. (**a**) Schematic illustration of SMRT sequencing. (**b**) Schematic illustration of bisulfite-based methylation sequencing (BS-seq). (**c**) Schematic diagram of methylome sequencing by enzymatic conversion. (**d**) Illustration of nanopore sequencing.

Among the various DNA modifications, 6mA is the most widely distributed modification in bacteria. In early studies, 6mA in bacterial genomes was mapped via R-M restriction endonucleases, revealing its essential role in regulating bacterial virulence (11). The study of 6mA in bacteria has entered a new era with the advent of SMRT sequencing (Fig. 1a). SMRT sequencing enables the simultaneous detection of nucleotides and all three major types of DNA methylation in prokaryotes via polymerase kinetics, as indicated by the interpulse duration. The signal-to-noise ratio exhibits high sensitivity for 6mA, moderate sensitivity for 4mC, and low sensitivity for 5mC (12). In 2012, Fang’s group obtained the first bacterial 6mA epigenome using SMRT sequencing (13); subsequently, thousands of bacterial epigenomes were decoded.

Bisulfite-based methylation sequencing (BS-seq) is considered the gold standard method for detecting 5mC (Fig. 1b). Upon treatment with bisulfite, unmethylated cytosine in DNA is converted to uracil, and then uracil is converted to thymine by polymerase chain reaction amplification. Unlike unmethylated cytosines, methylated cytosines are resistant to Cytosine (C) to Uracil (U) conversion (14, 15). In WGBS, high-throughput sequencing technology is employed to detect methylation across the genome after bisulfite treatment. This method is used to investigate differences in DNA methylation among different cell types, tissues, or stages of development. However, bisulfite treatment can induce DNA degradation, which results in DNA loss and biases in sequencing data. To overcome these defects, Dai et al. (16) developed a technique called ultrafast bisulfite sequencing (UBS-Seq) by optimizing the technical conditions. In UBS-Seq, the reaction time is reduced and DNA damage is minimized by increasing the temperature and concentration of bisulfite, which can be achieved by replacing sodium bisulfite with ammonium salt. Moreover, UBS-Seq results in less background noise than traditional BS-seq technology does when low-output samples are sequenced (16). Vaisvila et al. developed an EM-seq method in which TET2 and T4-BGT convert 5mC and 5hmC into 5caC and 5gmC, respectively, which are then deaminated by APOBEC3A, converting unmethylated cytosine to uracil in an enzymatic manner (17). EM-seq is believed to not induce DNA damage, which is a limitation of BS-seq (18) (Fig. 1c).

Despite these advances, challenges remain in differentiating between 4mC and 5mC, especially in bacteria, where the proportion of 4mC is greater. The emergence of nanopore sequencing has allowed the detection of all three types of DNA methylation via the measurement of current fluctuations during the translocation of DNA obstructing the nanopore (19, 20) (Fig. 1d). However, some challenges still need to be addressed. For example, the accuracy of a model trained to identify methylation sites is constrained by its training data, which limits its sensitivity and ability to detect methylation sites (21).

Recent advancements in understanding the mechanisms underlying the pathogenicity of bacterial pathogens have relied on the methods for DNA methylation sequencing described above. BS-seq/UBS-Seq and EM-seq are suitable for fragmented DNA samples, whereas SMRT and nanopore sequencing can be used for long-read sequencing of DNA. SMRT sequencing has the highest sensitivity for detecting 6mA modifications, moderate sensitivity for detecting 4mC modifications, and the lowest sensitivity for detecting 5mC modifications. In contrast, nanopore sequencing has greater accuracy for detecting 5mC modifications than for detecting 4mC and 6mA modifications. We believe that more precise and effective sequencing methods will be developed in the future, which will increase our understanding of the role of DNA methylation in bacterial pathogenesis. Such progress will be instrumental in the development of antibacterial agents.

## THE INVOLVEMENT OF DNA METHYLATION IN ANTIBACTERIAL DRUG RESISTANCE

Antimicrobial resistance (AMR) has become a principal cause of increasing infection rates and mortality. According to Ranjbar et al., approximately 4.95 million deaths were associated with AMR worldwide in 2019 (2). It is generally accepted that AMR arises from the expression of antibiotic resistance genes; however, the regulatory mechanisms governing the expression of these genes and their impact on AMR are not yet completely understood. In recent years, increasing evidence has demonstrated that DNA methylation regulates drug resistance in bacteria, with both DNA MTases from the R-M system and orphan MTases participating in this regulatory process (22).

**Fig 2:**
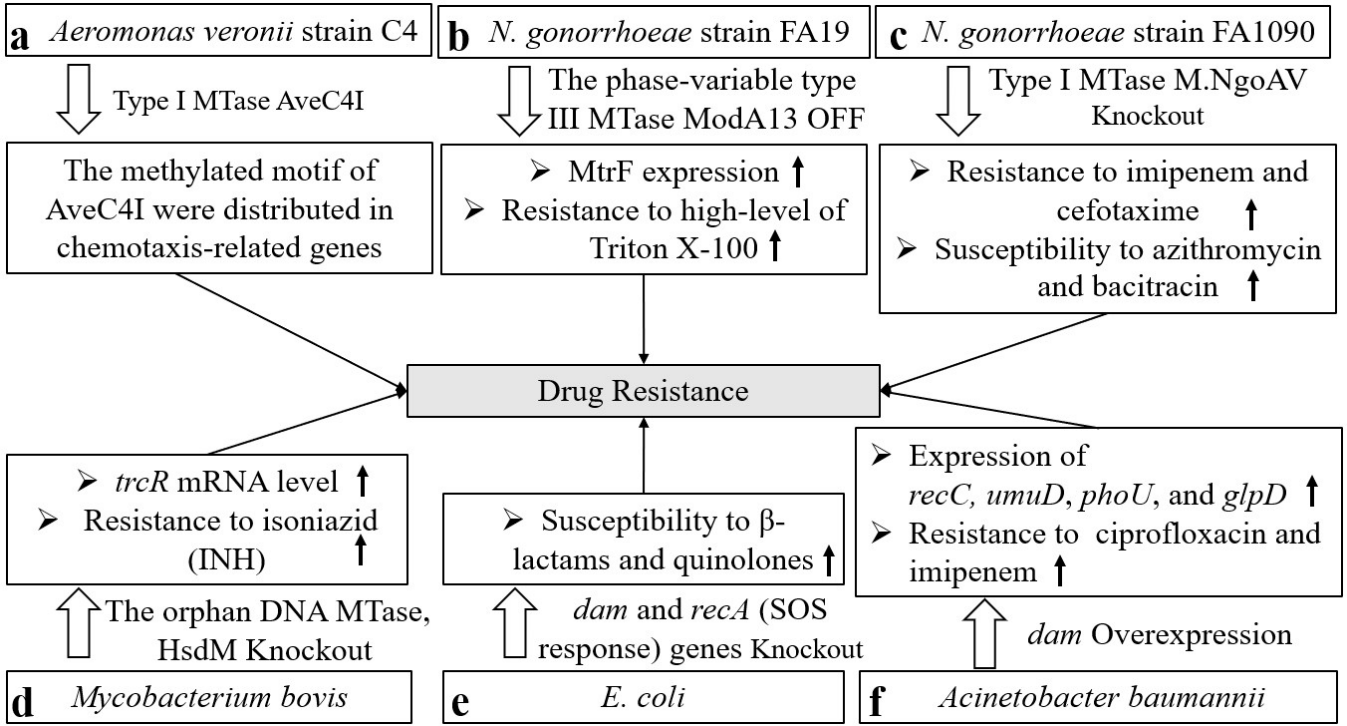
The involvement of DNA methylation in antibacterial drug resistance. (a) Type I MTase AveC4I regulates expression of chemotaxis-related genes in *Aeromonas veronii*. (b) When ModA13 is off, the expression of MtrF is upregulated, which mediated high levels of Triton X-100 resistance in *Neisseria gonorrheae.* (c) M.NgoAV knockout increases resistance to imipenem and cefotaxime, but decreases resistance to azithromycin and bacitracin in *Neisseria gonorrheae*. (d) In *Mycobacterium bovis*, the knockout of the orphan DNA MTase HsdM decreases susceptibility to isoniazid treatment by increasing *trcR* mRNA levels. (e) Knockout of the *dam* and *recA* (SOS response) genes significantly increases the susceptibility of *E. coli* to β-lactams and quinolones. (f) When *Acinetobacter baumannii* is exposed to ciprofloxacin and imipenem, the mRNA expression of *dam* and persister cell-related genes, such as *recC, phoU, glpD* and *umuD* are upregulated.

The data show that the MTases linked to drug resistance vary across different pathogens. In *Aeromonas veronii*, the methylated motif of the type I MTase AveC4I is distributed among response regulators in the chemotaxis system, such as *cheY and cheB*, which affect the drug resistance of *A. veronii* (23) (Fig. 2a); however, in *Neisseria gonorrhoeae* strain FA19, when the phase-variable type III MTase ModA13 is off, MtrF (a membrane protein that regulates antimicrobial resistance) is upregulated, which induces resistance to high levels of Triton X-100, and the bacteria also forms a dense and thick biofilm (24) (Fig. 2b). The type I methyltransferase M.NgoAV also regulates drug resistance in *N. gonorrhoeae* FA1090, and M.NgoAV knockout increases resistance to imipenem and cefotaxime, but decreases resistance to azithromycin and bacitracin (25) (Fig. 2c). Moreover, changes in the expression of ModA11 and ModA12, the cognate MTases of ModA13, affect bacterial susceptibility to ceftazidime and ciprofloxacin in *Neisseria meningitidis,* but a well-characterized mechanism is lacking (26). In *Streptococcus suis*, another type III DNA MTase, ModS2, affects ampicillin resistance by regulating the expression of relevant resistance proteins (27). Additionally, ModA2, ModA5, and ModA10 influence antibiotic resistance in *Haemophilus influenzae* (28). Nahar et al. reported that a cytosine-specific type III DNA MTase, ModP, regulates antibiotic resistance in *Actinobacillus pleuropneumoniae,* but the mechanisms involved are still unclear (29). These findings reveal the regulation of drug resistance by different MTases within bacterial R-M systems.

In addition to MTases in R-M systems, orphan MTases also contribute to bacterial drug resistance. For example, Hu and coworkers demonstrated that in *Mycobacterium bovis*, the absence of the orphan DNA MTase HsdM decreases susceptibility to isoniazid treatment by increasing *trcR* mRNA levels (*trcR* is thought to be a crucial regulator of the reactivation process), but the mechanisms still require further study (30) (Fig. 2d). Other well-known orphan MTases, such as Dcm and Dam, have been reported to increase the resistance of *Escherichia coli* MG1655 to antibiotics (31). Furthermore, synergistic knockout of the *dam* and *recA* (SOS response) genes significantly increases the susceptibility of *E. coli* to β-lactams and quinolones (32) (Fig. 2e). This was also demonstrated by Cohen and her group, who reported that the loss of adenine methylation at GATC sites (the target of Dam methylase) leads to the upregulation of error-prone polymerase IV and that the methyl-dependent mismatch repair pathway has deleterious effects under drug stress (33). In *E. coli* UTI89, *dam* mutants present significant defects in persister formation due to the downregulation of transcriptional regulators associated with bacterial resistance to antibiotics (34, 35). Similar findings were reported for *Acinetobacter baumannii* by Kim et al.; when *A. baumannii* is exposed to ciprofloxacin and imipenem, the mRNA expression of *dam* and persister cell-related genes, such as *recC, umuD* (essential for DNA repair during the SOS response), *phoU* (negatively regulates phosphate metabolism and plays roles in persister switching), and *glpD* (associated with energy metabolism), is upregulated (36) (Fig. 2f).

These findings demonstrate that DNA MTases and corresponding methylation sites related to drug resistance differ among different pathogens, highlighting the potential of MTases as promising targets for combating drug resistance.

## INFLUENCE OF DNA METHYLATION ON THE EXPRESSION OF BACTERIAL VIRULENCE GENES

It is generally accepted that the virulence of pathogens results from the expression of virulence factor genes. Virulence factors include adhesion molecules, such as type 1 fimbrial adhesin (37). Bacterial exoenzymes and toxins can be involved in the invasion step during pathogenesis (38); for example, bacterial pathogens secrete coagulase, an exoenzyme, to induce blood clotting and evade the immune system of the host (39). Capsule and bacterial proteases protect pathogens from phagocyte ingestion (40, 41), and antigenic variation helps pathogens evade detection by the immune system of the host (42). Various studies have shown that DNA methylation regulates these virulence factors.

**Fig 3:**
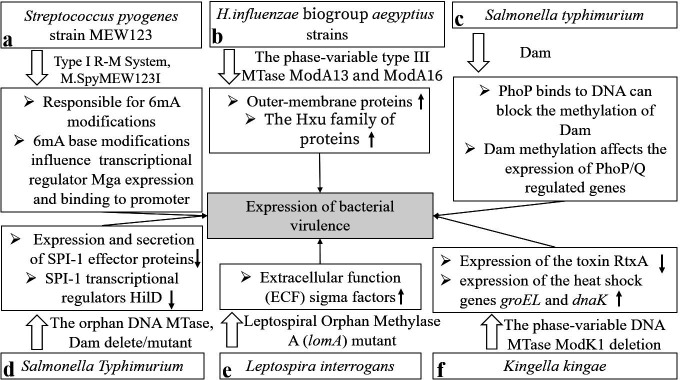
Influence of DNA methylation on the expression of bacterial virulence genes. (a) In *Streptococcus pyogenes*, 6mA modification, mediated by MTase SpyMEW123I, regulates the expression and binding of Mga, which activates multiple virulence factors expression. (b) ModA13 and ModA16 upregulate the expression of virulence factors, such as outer-membrane proteins and Hxu family proteins, in *H. influenzae* group *aegyptius*. (c) Dam mutants impact *Salmonella typhimurium* colonization at deep-tissue sites, and Dam regulates the expression of PhoP/PhoQ-regulated genes, contributing to virulence regulation. (d) In *Salmonella typhimurium,* the absence of Dam impairs the secretion of SPI-1 effector proteins and Dam mutants lower the expression of SPI-1 by modulating the posttranscriptional activity of the transcription factor HilD. (e) Mutants of *lomA* lead to dysregulation of extracellular function (ECF) sigma factors, whereas the overexpression of these sigma factors inhibits *Leptospira interrogans* toxicity. (f) In *Kingella kingae*, The deletion of *modK1* downregulates the expression of the toxin RtxA and upregulates the expression of the heat shock genes *groEL* and *dnaK*.

MTases associated with the R-M system were demonstrated to play crucial roles in virulence regulation. It has been reported that the MTase SpyMEW123I in *Streptococcus pyogenes* strain MEW123 mediates 6mA modification, which regulates the expression and binding of Mga. Mga is a master transcriptional activator of multiple virulence factors crucial for the survival of *Streptococcus pyogenes* in neutrophils (43, 44) (Fig. 3a). *Haemophilus* can colonize the respiratory tracts of patients, causing chronic bronchitis and further aggravating disease (45). ModA13 and ModA16 upregulate the expression of virulence factors, such as outer membrane proteins and Hxu family proteins, in *H. influenzae* group *aegyptius* The two MTases methylate the GCAC^m6^A and GGRC^m6^A motifs, respectively. However, the specific regulatory pathways directly involved in this process remain unclear (46) (Fig. 3b).

Orphan MTases also contribute to regulating virulence gene expression. Among orphan MTases, the depletion or overexpression of Dam is important for bacterial virulence. As reported decades ago, Dam mutants impact *Salmonella typhimurium* colonization at deep-tissue sites. Mechanistically, PhoP (a DNA-binding protein in the PhoP/PhoQ two-component signal transduction system) binds to DNA to block the methylation of Dam, and Dam regulates the expression of PhoP/PhoQ-regulated genes, thereby contributing to virulence regulation(11) (Fig. 3c). In some strains of *Salmonella*, the depletion of Dam regulates virulence through Salmonella pathogenicity islands (SPIs). In *Salmonella typhimurium,* the absence of Dam impairs the secretion of SPI-1 effector proteins such as SipA, SipB, SipC (47), and SopA (48). Moreover, in *S. typhimurium*, Dam mutants lower the expression of SPI-1 by modulating the post-transcriptional activity of the transcription factor HilD (49) (Fig. 3d). Furthermore, in *S. typhimurium*, Dam inhibits the transcriptional activation of *traJ* (TraJ is an activator of the *tra* operon) by competitively binding to the GATC site on the virulence plasmid gene with a leucine response regulatory protein (Lrp) (50). In *Yersinia pseudotuberculosis,* the overexpression of Dam attenuates virulence by decreasing the temperature-dependent ectopic secretion of the virulence protein Yops (51).

*Leptospira interrogans* has been demonstrated to have a robust ability to survive in external environments, posing a potential risk to humans and causing zoonotic diseases (52). Previous studies have suggested the existence of 4mC modification in the *Leptospira* genome (53), indicating that this modification is mediated by the conserved orphan MTase A (LomA) (54). Mutants of *lomA* lead to dysregulation of extracellular function sigma factors, whereas the overexpression of these sigma factors inhibits bacterial toxicity (55) (Fig. 3e). In *Kingella kingae*, the phase-variable DNA MTase ModK1 controls the expression of virulence-related genes and suppresses the host immune response by inhibiting the release of proinflammatory factors such as interleukin-8 (IL-8). The deletion of *modK1* downregulates the expression of the toxin RtxA and upregulates the expression of the heat shock genes *groEL* and *dnaK* (56) (Fig. 3f). A study comparing classic and hypervirulent *Klebsiella pneumoniae* revealed that hypervirulent strains present higher levels of adenine (GATC) and cytosine (CCWGG) methylation in multiple pathogenicity-related genes (57). These findings indicate that DNA methylation plays an essential role in both the survival and high virulence of *K. pneumoniae*. A previous study by Mehling revealed that the absence of DNA methylation partially attenuates the virulence of *K. pneumoniae*, while high doses of bacterial infection restore virulence to some extent (58). This finding suggests that DNA methylation, along with other factors, contributes to the regulation of bacterial virulence.

## THE IMPACT OF DNA METHYLATION ON BACTERIAL BIOFILM FORMATION, ADHESION, AND MOTILITY

Bacterial phenotypic properties, including biofilm formation, adhesion, and motility, are central to bacterial pathogenicity. Many studies have recently reported that DNA methylation affects the biological structures of bacteria, including biofilms, fimbriae, and flagella, which are closely related to bacterial adhesion, motility, and invasiveness in host target cells, significantly impacting bacterial pathogenicity. Dam seems to play a vital role in these phenomena, indicating that these compounds are promising targets for the development of antibacterial agents.

**Fig 4:**
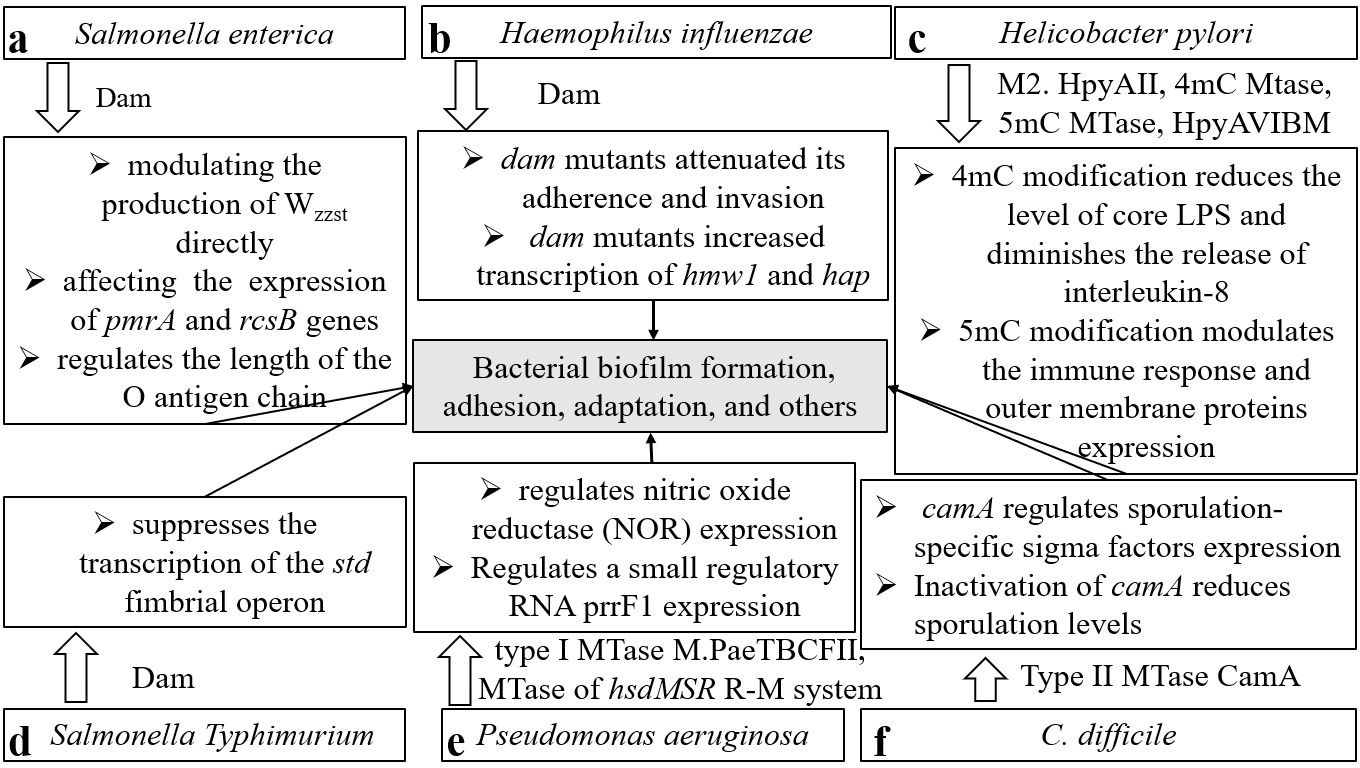
The role of DNA methylation in bacterial biofilm formation, adhesion, motility, and bacterial adaptation. (a) In *Salmonella enterica*, Dam regulates the length of the O antigen chain by modulating the production of Wzz_st_ directly or by affecting the expression of the RcsB and PmrA proteins, plays a significant role in biofilm formation. (b) In *H. influenzae*, *dam* mutants attenuated bacterial adhesion and invasion via increases in the expression of *hmw1* and *hap*. (c) In *Helicobacter pylori,* the N4-methylation-related cytosine MTase M2. HpyAII reduces the level of core LPS and diminishes the release of interleukin-8, and 5mC MTase *HpyAVIBM* induce the production of IL-8 in host AGS cells. (d) Dam suppresses the transcription of the *std* fimbrial operon in *S. typhimurium*, and modulated fimbriae synthesis or biofilm formation. (e) The type I MTase M.PaeTBCFII is closely related to nitric oxide reductase (NOR) expression and to influence bacterial denitrification in *Pseudomonas aeruginosa,* and 6mA DNA MTase of the *hsdMSR* R-M regulates small regulatory RNA *prrF1* expression, which contributes to iron metabolism. (f) In *Clostridium difficile*, type II 6mA solitary DNA MTase CamA primarily involves sporulation efficiency through regulating sporulation-specific sigma factors expression.

In *Salmonella enterica*, for example, Dam contributes to phenotypic heterogeneity (59). Specifically, Dam regulates the length of the O antigen chain by modulating the production of Wzz_st_ directly or by affecting the expression of the RcsB and PmrA proteins, whereas RcsB and PmrA bind to the *wzz_st_* promoter and initiate its transcription through the PmrA/PmrB and RcsC/YojN/RcsB systems, respectively (Fig. 4a). This regulation subsequently modifies the structural composition of lipopolysaccharide (LPS), which plays a significant role in biofilm formation (60, 61). In *E. coli,* Dam positively regulates the production of antigen 43 (Ag43), an outer membrane protein, whereas OxyR negatively regulates it by competitively binding to GATC sites with Dam. It dynamically modulates *agn43* expression (62–64), which facilitates biofilm formation under specific conditions (such as in glucose-containing medium) and influences cell interactions. In *Actinobacillus pleuropneumoniae* strain AP76, a type III DNA MTase, ModP1, also promotes biofilm formation and increases growth rates (29).

Notably, bacterial adhesion can be regulated by a series of MTases. In *H. influenzae*, Jarisch et al. experimentally demonstrated that insufficient *dam* expression reduces virulence both *in vivo* and *in vitro* by modulating bacterial adhesion and invasion via increases in the expression of *hmw1* and *hap* (genes involved in adherence and invasion) (65) (Fig. 4b). In multiple cases, this regulation of adhesion is accomplished through the modulation of fimbriae synthesis or biofilm formation. Specifically, Dam suppresses the transcription of the *std* fimbrial operon in *S. typhimurium* (66) (Fig. 4d) and modulates fimbrial expression in *E. coli* by regulating the transcription level of the *papI* gene (a local regulator of Pap phase variation) (67). In enterohemorrhagic *E. coli* O157:H7, deletion of Dam increases bacterial adhesion to mammalian cells by increasing the level of bacterial outer membrane adhesins and the production of the actin pedestal (68, 69). Dam also exhibits diverse regulatory effects in other *E. coli* strains. For example, in enteroaggregative *E. coli*, Dam and ferric uptake regulator (Fur) compete for binding to a certain DNA sequence within the −10 transcriptional element, thereby regulating the transcription of type VI secretion system genes (70). In *Helicobacter pylori,* the absence of M2.HpyAII, the only N4-methylation-related cytosine MTase responsible for the methylation of the TCTTC sequence, reduces the level of core LPS and diminishes the release of interleukin-8 from human gastric adenocarcinoma cells (71). This reduction facilitates the adhesion of *H. pylori* to AGS cells (a human gastric adenocarcinoma cell line) and triggers an inflammatory response. In contrast, mutations in the orphan 5mC MTase *HpyAVIBM* induce the production of IL-8 in host AGS cells, and HpyAVIBM-targeted genes, such as outer membrane proteins, may be upregulated in *hpyAVIBM* mutant strains and induce an immune response in AGS cells (72) (Fig. 4c). Additionally, in *H. pylori* strains SS1 and AM5, HpyAVIBM modulates the transcription levels of fucosyltransferases, such as FutA, FutB, and FutC, which are responsible for the synthesis or active induction of Lewis antigens and the structural modification of fucose sugars to form LPS (72–74). Another highly conserved 5mC MTase in *H. pylori strain* J99, M.Hpy99III, affects bacterial adhesion, probably also by impacting the production of outer membrane proteins (75). A nontypeable *H. influenzae* strain exhibits comparable phenotypic changes regulated by a 6mA MTase (ModA). This enzyme influences the expression of high-molecular-weight proteins, which are involved in bacterial biofilms and the mediation of immune escape (28). Garai reported that phase variation in ModA affects bacterial adhesion, partially through adhesion-associated substances such as the adhesin proteins E and P4 (76).

In addition to affecting biofilm formation and adhesion, DNA methylation has a profound effect on bacterial motility. In *S. Typhimurium*, the murein lipoprotein (Lpp), encoded by the genes *lppA* and *lppB*, is involved in motility and cytotoxicity (77). Pucciarelli and coworkers reported that the absence of Dam impedes the binding of membrane proteins, including murein lipoprotein and peptidoglycan, facilitating the efflux of bacterial substances and destabilization of the bacterial envelope (78). DNA methylation also influences the motility of bacteria by regulating flagella formation. In *H. pylori*, the interactions among different MTases add to the complexity of phenotypic regulation by DNA methylation (79). ModH5, a type III 6mA DNA MTase, increases the expression of the flagellin A (*flaA*) gene in *H. pylori* P12 either directly or indirectly by the regulatory factor *fliK* (80). Kumar reported that the HpyAVIBM system regulates the expression of flagellar genes such as *rpoN* and *fliR* while also suppressing the expression of cytotoxin-associated proteins, including CagA and VacA, in *H. pylori* strains AM5 and SS1 (72). Notably, the methylation pattern of *H. pylori* is dynamically regulated within the acidic environment of the host by the two-component system ArsRS and the type I 6mA DNA MTase HsdM1. During this process, ArsRS controls the expression of *hsdM1* (81).

The regulatory effects of DNA methylation on bacterial biofilms, motility, and adhesion have direct implications for bacterial pathogenicity. By modulating the key phenotypic traits of bacterial pathogens, DNA methylation influences their ability to infect host cells, evade the immune system, and establish chronic infections. As highlighted by various studies on pathogens such as *S. enterica, E. coli, H. pylori*, and *A. pleuropneumoniae*, DNA methylation plays a central role in the regulation of pathogenicity-related traits.

## THE ROLE OF DNA METHYLATION IN BACTERIAL ADAPTATION

Pathogens encounter a variety of challenges, such as oxidative stress, unfavorable temperatures, and hypoxic conditions. The adaptive capacity of pathogens is crucial for their survival. Epigenetic modifications are believed to play an important role in facilitating their adaptation.

Costeira et al. characterized the changes in the methylome and transcriptome of *Porphyromonas gingivalis* W50 under conditions of excess or limited hemin availability. The results revealed that excess hemin induced the expression of virulence determinants, with Dam and Dcm regulating the expression of such determinants related to lactate utilization and the ATP-binding cassette transporter (82). Additionally, Dam is known to regulate the transcription of antioxidant genes in *Salmonella*, playing an indispensable role in the adaptation of *S. typhimurium* to hydrogen peroxide stress (83). Dam-mediated methylation significantly increases the serum resistance of *Klebsiella pneumoniae* genotype K1 (84). Dam-mediated methylation contributes to the adaptation of pathogens to temperature changes. Vân Hofwegen and coworkers compared the dam-mediated methylation levels of *Yersinia enterocolitica* at 22°C and 37°C and reported that differentially methylated sites in the promoter regions of LysR-type transcription regulator control the expression of genes related to metabolism and virulence (85). The 6mA MTase MamA favors the survival of *Mycobacterium tuberculosis* under hypoxic conditions; mechanistically, MamA modulates hypoxia-related gene expression through the methylated motif overlapping with the sigma factor binding site (86), and HsdM, another 6mA MTase found in *Mycobacterium bovis*, also appears to play an important role in bacterial survival under hypoxic conditions (30). In a chronically adapted *Pseudomonas aeruginosa* strain (clinical strain TBCF10839), the type I MTase M.PaeTBCFII was found to be closely related to nitric oxide reductase expression and to influence bacterial denitrification (87) (Fig. 4e). Similarly, a 6mA DNA MTase of the *hsdMSR* R-M system was identified in *P. aeruginosa* PAO1; it plays a role in the oxidative stress resistance of this opportunistic pathogen by regulating small regulatory RNA *prrF1* expression, and *prrF1* contributes to iron metabolism (88) (Fig. 4e). Sporulation is an important biological process for bacterial adaptation. It has been reported that DNA methylation regulates sporulation in *Clostridium difficile*. As a predominant cause of nosocomial diarrhea infections and colitis worldwide, hypervirulent strains of *C. difficile* pose a significant threat to public health (89). The type II 6mA solitary DNA MTase CamA, which acts at the CAAAAA locus, is ubiquitous and highly conserved among *C. difficile* genomes. Deficiency of *camA* leads to decreased sporulation efficiency *in vitro*, primarily due to the reduced expression of some sporulation-related factors, such as sporulation-specific sigma factors (90). Spore formation in *C. difficile* primarily involves a complicated cascade of events involving the essential protein Spo0A (91, 92) and specific sigma factors (92) (Fig. 4f). Among these regulatory factors, sigma F might be the initial transcription factor activated (90), as proven by Jones and colleagues in their research on *Clostridium acetobutylicum* (93). The effect of methylation on bacterial sporulation has also been demonstrated in *Streptomyces coelicolor* A(3)2 M145, although it involves 5mC rather than 6mA (94). These findings reveal that MTases have broad regulatory functions in the adaptation of pathogens to various stressors, including oxidative stress, temperature changes, and hypoxic stress. The inactivation of MTases could be a promising strategy to prevent bacterial infections.

## CONCLUSION AND PERSPECTIVE

**Fig 5:**
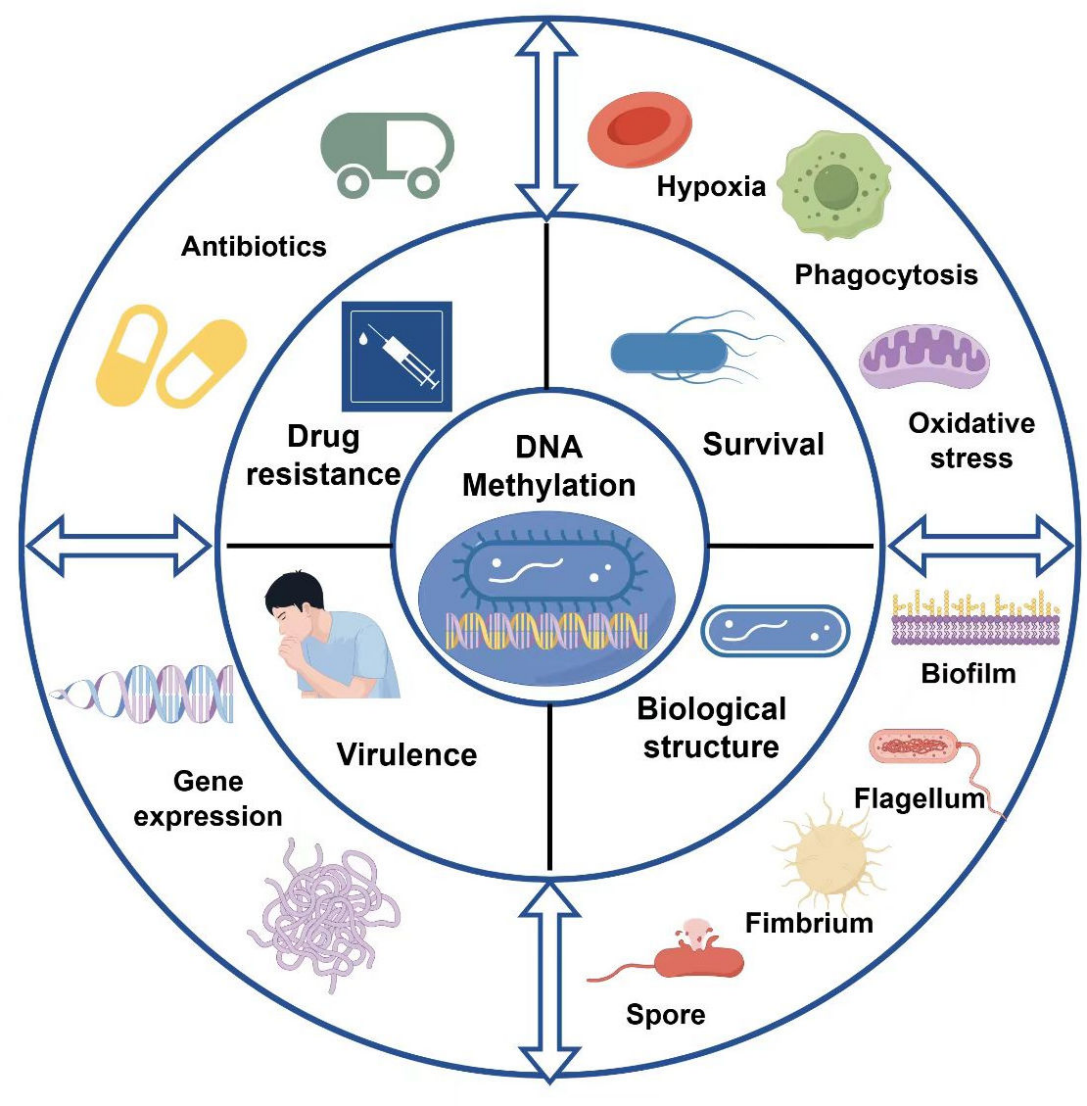
The role of DNA methylation in regulating the pathogenicity of pathogens. DNA methylation controls bacterial resistance, particularly to antibiotics; it influences a pathogen’s adaptability to environmental stressors such as hypoxia, oxidative stress, and phagocytosis; it regulates the expression of bacterial virulence genes; and it modulates bacterial biological functions, including biofilm formation, adhesion, and motility.

Although bacteria have heterochromatin-like structures (95), bacteria lack the post-translational modifications observed in eukaryotes, and epigenetic modifications in bacteria are primarily DNA methylation, mainly 5mC, 4mC, and 6mA. Significant progress has been made in understanding the interplay between MTases and regulatory factors that govern the transcriptional regulation of virulence-related gene operons. Comprehensive mapping of the epigenomes of opportunistic pathogens in both nonpathogenic and pathogenic states is valuable for identifying critical epigenetic regulatory factors. In this review, we analyze the existing research on the role of DNA methylation in regulating the pathogenicity of pathogens for the first time. We discuss recent advancements in technologies for DNA methylation sequencing of bacteria, summarize the contributions of DNA methylation to bacterial drug resistance and adaptive responses, and further discuss the impact of DNA methylation on the regulation of virulence gene expression, biofilm formation, colonization, and motility (Fig. 5). We hope to identify the key MTases and related methylation sites or motifs that regulate the pathogenicity of pathogens, thus offering a novel direction for the prevention and treatment of bacterial infections from the perspective of epigenetics.

The studies highlight the diversity of DNA MTases associated with pathogenicity in a wide range of pathogenic bacteria. Each pathogen has its own unique MTases that contribute to its pathogenicity, and even within the same bacterial family, the key MTases differ among different species. However, a universal DNA MTase with broad regulatory effects on pathogenicity against pathogens has not yet been identified. Additionally, compared with the development of 5mC sequencing methods, the development of 6mA and 4mC sequencing methods has lagged far behind. Although third-generation sequencing technology makes it possible to obtain bacterial 6mA and 4mC methylomes, high costs and false-positive rates might hinder the wide adoption of such sequencing technologies. Therefore, more precise and effective methods are still needed in the future.

Alterations in DNA methylation may disrupt the regulation of the bacterial virulence balance, potentially impacting bacterial structural integrity or physiological function. DNA methylation is critical for competitive survival and pathogenicity within bacterial populations, and DNA methylation sites may serve as new pathogenic biomarkers for opportunistic pathogens. This epigenetic marker has broad prospects for application in disease prevention, diagnosis, and treatment. In the future, is it possible to identify the key DNA MTases in pathogens? What are the key methylation sites related to pathogenicity? Is it possible that DNA methylation sites can serve as biomarkers for predicting pathogenicity? To date, most studies have focused on identifying MTases, but the role of demethylases in bacterial pathogenicity remains poorly understood. These issues require further in-depth research.
